# Visual field–based reaction time as a novel indicator for early detection of mild cognitive impairment

**DOI:** 10.1038/s41598-025-32034-6

**Published:** 2025-12-11

**Authors:** Yoshiki Tamaru, Shin Inada, Norio Ideguchi, Shohei Kagino, Yuki Katsuhara, Yasuhiro Higashi

**Affiliations:** 1 Department of Occupational Therapy, Faculty of Health Sciences, Kyoto Tachibana University, 34 Oyake yamada-cho, Yamashina-ku, Kyoto, 607-8175 Japan; 2 Department of Clinical Engineering, Faculty of Medical Technology, Morinomiya University of Medical Sciences, 1-26-16 Nankokita, Suminoe-ku, Osaka, 559-8611 Japan; 3 Department of Acupuncture, Faculty of Health Sciences, Morinomiya University of Medical Sciences, 1-26-16 Nankokita, Suminoe-ku, Osaka, 559-8611 Japan; 4 Department of Occupational Therapy, Faculty of Rehabilitation, Morinomiya University of Medical Sciences, 1-26-16 Nankokita, Suminoe-ku, Osaka, 559-8611 Japan; 5 Department of Rehabilitation, Faculty of Health Sciences, Naragakuen University, 3-15-1 Nakamachi, Tomigaoka, Nara, Nara 631-0003 Japan

**Keywords:** Mild cognitive impairment, Peripheral vision, Useful field, Reaction time, Biomarkers, Neurology, Neuroscience, Psychology, Psychology

## Abstract

Early detection of mild cognitive impairment (MCI) is vital for timely intervention to delay or prevent progression to dementia. Gaze behavior analysis has been shown to differentiate individuals with MCI from cognitively healthy older adults. This study aimed to examine visual processing differences between cognitively healthy older adults and those with MCI, focusing on central and Useful Field of View (UFOV) tasks. Participants completed a central visual field task and a UFOV task. Reaction times, omission and commission errors, and visual orienting frequency were measured. Group comparisons were conducted. For variables showing significant differences, receiver operating characteristic curve analysis evaluated discriminatory accuracy and optimal cutoff values. No significant group differences emerged in the central task. In the UFOV task, patients with MCI demonstrated significantly slower reaction times than controls. The optimal UFOV reaction time cutoff was 598.1 ms, with 90.3% sensitivity, 72.1% specificity, and an area under the curve of 0.841. Older adults with MCI exhibit delayed visual processing under UFOV conditions. Reaction time in the UFOV task may serve as a sensitive, practical behavioral marker for early MCI detection.

## Introduction

The global aging of the population has led to a substantial rise in the number of older adults with dementia, placing growing burdens on healthcare systems and social security infrastructures worldwide^1^. To delay or prevent dementia onset, early detection and intervention during the prodromal stage—mild cognitive impairment (MCI)—are essential ^2,3^. MCI represents an intermediate stage between normal cognitive aging and dementia, marked by measurable deficits in memory or other cognitive domains, while daily functional independence remains largely preserved. Non-pharmacological interventions, including lifestyle modification, aerobic exercise, and cognitive training, have shown promise in slowing progression when applied during the MCI stage ^4–6^.

Recently, disease-modifying therapies such as lecanemab (LEQEMBI)^7^ and donanemab^8^ have demonstrated efficacy in slowing cognitive decline and, in some cases, improving cognitive function when initiated during MCI. Consequently, timely and accurate identification of individuals with MCI is crucial to optimize the effects of both pharmacological and non-pharmacological treatments. However, conventional screening methods are often influenced by educational background and language proficiency, limiting their sensitivity in detecting early cognitive changes ^9^. This limitation highlights the need for practical, scalable, and sensitive tools capable of identifying early cognitive decline before functional impairments appear.

Beyond documented deficits in memory ^10,11^, attention^10,12,13^, and executive function^14,15^, emerging evidence suggests that subtle impairments in visual processing and attentional control may serve as early indicators of cognitive vulnerability ^16–20^. In visual cognition, the central visual field supports high-resolution object recognition, while the Useful Field of View (UFOV) enables rapid, accurate processing of peripheral visual information surrounding the fixation point ^21,22^. These functions are particularly susceptible to aging, and UFOV contraction has been linked to reduced attentional resources and increased cognitive load ^23^. Based on these findings, measuring reaction times to stimuli within the central visual field and UFOV has emerged as a promising method for detecting early cognitive decline in aging and MCI. Variations in visual response characteristics may reflect changes in processing speed and attentional resource allocation, offering sensitive behavioral markers of cognitive impairment.

In this study, we aimed to examine whether UFOV reaction time could serve as a sensitive and practical behavioral marker for distinguishing older adults with MCI from cognitively healthy peers. We hypothesized that reaction time in the UFOV condition, but not the central condition, would significantly differ between groups, and that UFOV-RT would demonstrate strong discriminatory accuracy compared to conventional cognitive screening tests.

Against this background, the present study extends prior UFOV work in several ways. First, we combine trial-level reaction time (RT) with concurrent head-mounted eye-tracking (omission/commission errors and visual orienting) within a single paradigm, providing a more fine-grained behavioral–oculomotor characterization than composite UFOV scores alone. Second, we evaluate diagnostic performance using logistic regression and receiver operating characteristic (ROC) analyses to derive probability-based thresholds and an explicit, clinically actionable RT cut-off in milliseconds. Third, we benchmark UFOV-RT against widely used cognitive screeners (MoCA, MMSE) to contextualize its incremental utility for early detection. Research question and hypotheses. We asked whether UFOV-RT can serve as a sensitive and practical behavioral marker to distinguish older adults with MCI from cognitively healthy peers. We hypothesized that (i) RT in the UFOV condition—but not in the central condition—would significantly differ between groups; and (ii) UFOV-RT would exhibit strong discriminatory accuracy, comparable to or exceeding conventional cognitive screening tests, as quantified by ROC analyses.

## Methods

### Study design and participants

This cross-sectional study was conducted between 2024 and 2025 and included older adults residing in medical and nursing care facilities. Participants were classified into two groups: those with MCI (MCI group) and cognitively healthy controls (HC group). Inclusion criteria for the HC group were a score of ≥ 26 on the Japanese version of the Montreal Cognitive Assessment (MoCA-J) and a score of 28–30 on the Japanese version of the Mini-Mental State Examination (MMSE-J). The MCI group included patients with MoCA-J scores ≤ 25 and MMSE-J scores 24–27 ^15^. Exclusion criteria included: visual impairments, regular use of corrective glasses, cerebrovascular disease, or other neurological or medical conditions likely to affect cognitive or visual performance. A priori power analysis was conducted using G*Power version 3.1.9.7 (Heinrich Heine University Düsseldorf, Germany) ^24^ to estimate the minimum sample size for logistic regression. Parameters included a two-tailed test, odds ratio of 3.0 (large effect), Pr (Y = 1 | X = 1) = 0.5, α = 0.05, power (1 − β) = 0.80, and two predictor variables. The proportion of variance explained by other predictors (R^2^ other X) was set between 0.0 and 0.1, resulting in a required sample size of approximately 70–80. A total of 79 participants were initially recruited. After excluding five individuals who met exclusion criteria, 74 patients were included in the final analysis: 43 in the HC group (mean age: 72.2 ± 2.9 years; 21 males, 22 females; MoCA-J: 28.1 ± 1.0; MMSE-J: 29.4 ± 0.7) and 31 in the MCI group (mean age: 73.4 ± 3.9 years; 18 males, 13 females; MoCA-J: 22.7 ± 1.3; MMSE-J: 25.7 ± 1.0). Participant characteristics are summarized in Table 1. The study protocol was approved by the Ethics Committee of Kyoto Tachibana University (Approval Number: 25–11). No financial incentives or compensation were provided. All methods were carried out in accordance with relevant guidelines and regulations. Informed consent was obtained from all individual participants included in the study.

**Table 1:** Participant characteristics.

	HC-group	MCI-group	t	df	*p*-value	Cohen’s d	95% CI	
							Lower	upper
n	43	31	–	–	–	–	–	–
Age	72.2 ± 2.9	73.4 ± 3.9	− 1.57	72	0.12	− .0369	− 0.83	0.10
MoCA-J	28.1 ± 1.0	22.7 ± 1.3	19.5	72	< 0.001	− 1.095	3.71	5.47
MMSE-J	29.4 ± 0.7	25.7 ± 1.0	18.0	72	< 0.001	− 1.071	3.41	5.08

*MoCA-J* Japanese version of the montreal cognitive assessment, *MMSE-J* Japanese version of the mini-mental state examination, Statistical analysis was performed using an independent samples t-test.

## Outcome measures

All assessments were conducted with participants seated in a stable, upright position. A high-precision eye-tracking system (View Tracker III; DITECT Co., Ltd., Tokyo, Japan)^15^ was used to measure gaze behavior. Visual stimuli were presented on a 23.8-inch external monitor (model PTFWJA, PRINCETON, Japan) positioned 1 m in front of the participant’s forehead at eye level. Two visual tasks were administered: a central visual field task and a UFOV task, each designed to assess different aspects of visual processing.

The central visual field task evaluated the ability to detect stimuli presented at the fixation point. The UFOV task assessed responses to stimuli located in the peripheral visual field surrounding the fixation point. For each task, the following variables were recorded:

Outcome measures included:

(1) Central reaction time (C-RT) and Useful-Field Reaction Time (U-RT), measured in milliseconds (ms); (2) central omission errors (C-OE) and useful-field omission errors (U-OE), defined as the number of missed responses to target stimuli; (3) central commission errors (C-CE) and Useful-Field Commission Errors (U-CE), defined as the number of false responses to non-target stimuli; and (4) visual orienting frequency (VOF), defined as the number of gaze shifts away from the central fixation point toward peripheral stimuli.

These variables were compared between the MCI and HC groups to assess group-level differences and evaluate their potential as behavioral markers of cognitive impairment.

### Stimulus conditions: visual reaction assessment system (VRAS)

Experimental tasks were developed using Microsoft Visual Studio Community 2022 and run on a personal computer (Let’s Note CF-FV; Panasonic Corporation, Japan) with Windows 11 Home. Stimulus presentation was controlled by a custom-built VRAS.

The two tasks were administered in separate blocks. To minimize potential carry-over effects, the central visual field task was always presented first, followed by the UFOV task. Each task required approximately 3–4 min, resulting in a total testing time of about 7–8 min. In the UFOV task, 80 trials were divided into four blocks of 20 trials each. On each trial, the target stimulus appeared randomly in one of the four peripheral locations (upper right, lower right, lower left, upper left), with 20 trials assigned to each location across the task. This design minimizes fatigue and allows for consistent task pacing.

Condition 1 (central visual field task): A red circular target (4 cm diameter) was displayed 20 times at the center of the screen. Participants were instructed to press a response button as quickly and accurately as possible when the target appeared.

Condition 2 (UFOV task): A black fixation point of the same size remained at the center of the screen. While maintaining gaze on the fixation point, red circular targets identical to those in Condition 1 were presented in one of four peripheral locations—upper right (45°), lower right (135°), lower left (225°), or upper left (315°)—each 20 cm from center. Each location included 20 randomized trials, totaling 80. Each stimulus was displayed for 2 s.

The C-RT task consisted of 20 trials and the UFOV task consisted of 80 trials. Although the total task duration differed between tasks, the timing parameters for each individual trial (stimulus presentation time, response window, and inter-trial interval) were identical across both tasks. Thus, the tasks were comparable in their trial-level temporal structure but not in total duration.

**Fig. 1:**
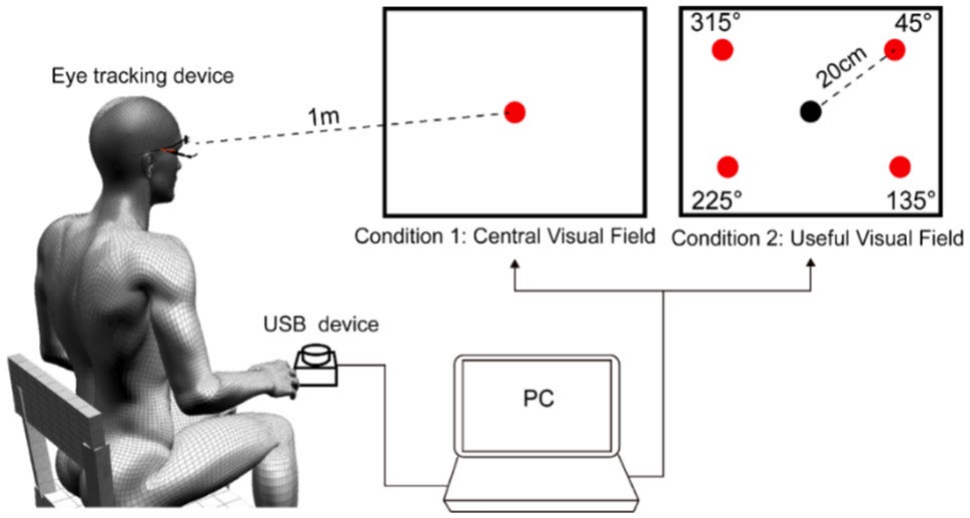
Experimental conditions of the Visual Reaction Assessment System (VRAS). Participants sat in an upright position and were instructed to press a response button upon detecting a red circular target. In Condition 1 (central visual field task), the target was presented at the center of the screen. In Condition 2 (useful field of view task), while maintaining gaze on a central black fixation point, targets appeared randomly at four peripheral locations (45°, 135°, 225°, and 315°), each positioned 20 cm from the center. All stimuli were 4 cm in diameter and presented for 2 s per trial. Each condition consisted of 20 trials with randomized timing.

Reaction time was recorded using a USB-connected response button (model USBSWP; TECHNOTOOLS, Japan). Reaction time was defined as the latency between stimulus onset and button press. In addition to reaction time, omission errors, commission errors, and—exclusively for Condition 2—VOF (i.e., number of gaze deviations from the fixation point) were recorded (Fig. 1).

### Eye-tracking apparatus and gaze data analysis

**Fig. 2:**
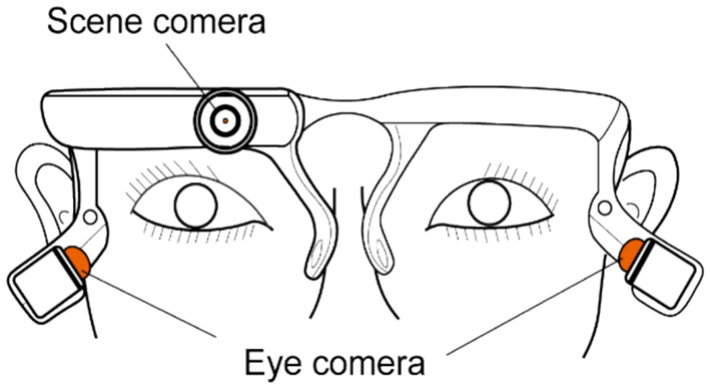
Experimental setup of the eye-tracking system. The device (View Tracker 3, Direct Co., Ltd., Tokyo, Japan) is equipped with a scene camera and two eye cameras, allowing simultaneous recording of gaze behavior during task performance.

Gaze behavior was continuously recorded using the View Tracker 3 (DITECT Inc., Tokyo, Japan), a lightweight head-mounted device (~ 40 g) equipped with a 200 Hz infrared eye camera and a 30 Hz scene camera to capture both eye position and the participant’s visual field. The device connected to a computer via USB, allowing synchronized data acquisition. Gaze data were processed using DITECT gaze analysis software (version 1.0518). Before the experimental tasks, standardized calibration was conducted individually to ensure accuracy (Fig. 2). Gaze data were analyzed using an area-of-interest (AOI)–based method. AOIs were predefined to match the spatial locations of target stimuli in each condition. The eye-tracking system was integrated with the VRAS platform, enabling real-time gaze behavior analysis under both visual conditions. In Condition 1, participants were required to fixate on the centrally presented target and respond promptly. In Condition 2, gaze accuracy was evaluated based on whether attention was incorrectly directed toward peripheral, non-target stimuli. Incorrect responses were recorded in both conditions and used as quantitative indicators of gaze-related error.

### Statistical analysis

All statistical analyses were conducted using IBM SPSS Statistics for Windows, version 30.0 (IBM Corp., Armonk, NY, USA). Group comparisons were performed for four primary outcome variables: reaction time, omission errors, commission errors, and VOF. The Shapiro–Wilk test assessed the normality of each variable. Reaction time met normality assumptions and was analyzed using an independent samples t-test. Omission errors, commission errors, and VOF violated normality assumptions and were analyzed using the Mann–Whitney U test. Variables that differed significantly between the MCI and HC groups in preliminary analyses were entered as predictors in a binomial logistic regression model, with group classification (HC = 0, MCI = 1) as the dependent variable. For predictors that were statistically significant in the logistic model, receiver operating characteristic (ROC) curve analysis evaluated classification performance. The area under the curve (AUC) was calculated to assess model accuracy, and optimal cutoff values were identified using the Youden index. A significance level of *p* < 0.05 was used for all tests. In addition, Pearson’s correlation analysis was conducted to examine the association between central RT (C-RT) and UFOV RT (U-RT).

A mixed-design ANOVA was conducted with task condition (C-RT vs. U-RT) as a within-subject factor and group (HC vs. MCI) as a between-subject factor. In addition, an ANCOVA was performed using C-RT as a covariate to examine group differences in U-RT while controlling for baseline response speed.

## Results

Group differences between older adults with MCI and cognitively healthy controls (HC) were examined using appropriate statistical procedures. Outcome variables included reaction time, omission errors, commission errors, and gaze-related indicators under central visual field and UFOV conditions.

Variables showing statistically significant between-group differences were further analyzed using binomial logistic regression to evaluate classification accuracy for MCI. Predictors that reached statistical significance in the regression model underwent ROC curve analysis to assess discriminatory performance. The AUC and optimal cutoff values were calculated using the Youden index.

### Results of between-group analyses

#### Reaction time

**Fig. 3:**
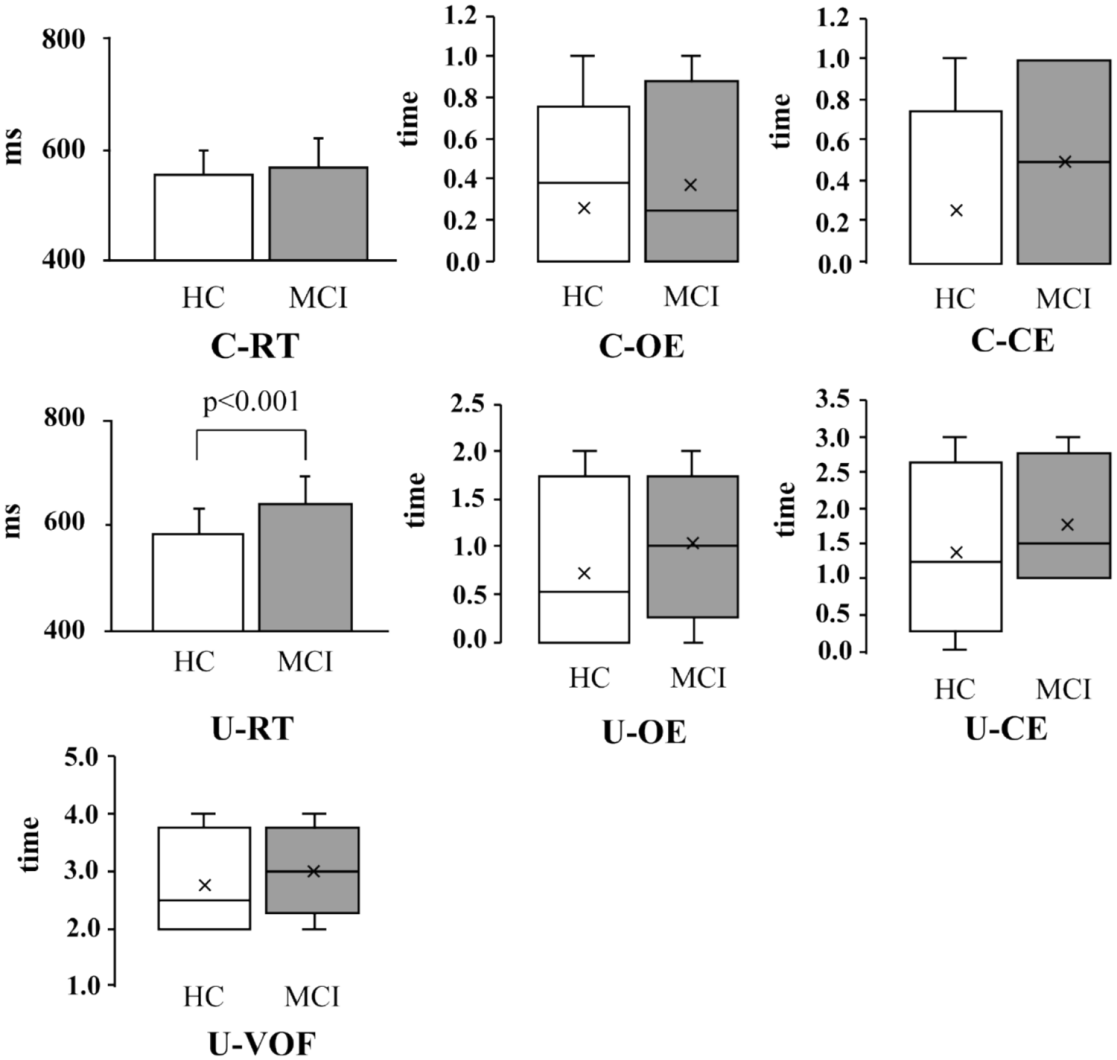
Boxplots comparing central and useful field measures between the MCI and HC groups. (**A**) Central Reaction Time (C-RT); (**B**) Useful-Field Reaction Time (U-RT); (**C**) Central Omission Errors (C-OE); (**D**) Useful-Field Omission Errors (U-OE); (**E**) Central Commission Errors (C-CE); (**F**) Useful-Field Commission Errors (U-CE); (**G**) Useful-Field Visual Orienting Frequency (U-VOF). Each box represents the interquartile range (IQR), with the horizontal line indicating the median and the “×” symbol indicating the mean. Whiskers represent the range of values excluding outliers. The MCI group tended to show slower response times and greater variability in error measures and gaze behavior compared to the HC group.

For central reaction time (C-RT), the HC group had a mean of 554.4 ms (SD = 46.6), and the MCI group had a mean of 568.5 ms (SD = 52.6). This difference was not statistically significant (t = − 1.22, p = 0.228, Cohen’s d = − 0.29). In contrast, for Useful-Field Reaction Time (U-RT), the HC group recorded a mean of 580.3 ms (SD = 44.9), while the MCI group had a significantly slower mean of 638.6 ms (SD = 38.4), indicating a significant group difference (t = − 5.84, *p* < 0.001, Cohen’s d = − 1.17) (Fig. 3). In addition, a significant positive correlation was observed between C-RT and U-RT (r = 0.545, 95% CI [0.358, 0.686], *p* < 0.001, n = 74).

A mixed-design ANOVA with task condition (C-RT vs. U-RT) as the within-subject factor and group (HC vs. MCI) as the between-subject factor revealed a significant Task × Group interaction, F(1,72) = 19.09, *p* < 0.001, ηp^2^ = 0.210. This indicates that the group difference in response time was disproportionately larger for U-RT than for C-RT.

To further examine whether the MCI–HC difference in U-RT could be explained by general response speed, an ANCOVA was conducted with U-RT as the dependent variable and C-RT as a covariate. The covariate was significant, F(1,72) = 34.04, *p* < 0.001, ηp^2^ = 0.324, and the group effect remained highly significant after controlling for C-RT, F(1,72) = 37.94, *p* < 0.001, ηp^2^ = 0.348.

#### Omission error

For central omission errors (C-OE), the median was 0.0 in both groups (interquartile range [IQR]: 0.0–0.0), showing no between-group variability. The difference was not statistically significant (*U* = 745.5, Z = 1.276, *p* = 0.202, *r* = 0.148). In the UFOV task (U-OE), the median was also 0.0 in both groups, but the MCI group had a wider IQR (0.0–0.5) than the HC group (0.0–0.0), indicating greater performance variability. Although not statistically significant (*U* = 790.0, Z = 1.517, *p* = 0.129, *r* = 0.185), this trend may suggest increased omission errors among older adults with MCI (Fig. 3).

#### Commission error

For central commission errors (C-CE), no significant group difference was observed (*U* = 705.0, Z = 0.557, *p* = 0.577, *r* = 0.065). In the UFOV task (U-CE), the MCI group showed slightly more errors (median = 1.0, IQR 0.0–1.5) than the HC group (median = 0.0, IQR 0.0–1.0), but the difference was not statistically significant (*U* = 792.0, Z = 1.464, *p* = 0.143, *r* = 0.170) (Fig. 3).

#### UFOV

In the UFOV task, the MCI group exhibited a slightly higher VOF (median = 3.0, IQR 2.0–3.0) compared to the HC group (median = 2.0, IQR 2.0–3.0). This difference approached but did not reach statistical significance (U = 833.0, *p* = 0.052, r = 0.230) (Fig. 3).

### Logistic regression analysis

Logistic regression analysis was conducted to determine whether U-RT could distinguish older adults with MCI from those who were cognitively healthy. As shown in Table 2, U-RT was a significant predictor (B = 0.03, SE = 0.01, Wald = 17.55, *p* < 0.001), with an odds ratio of 1.03 (95% CI 1.017–1.046). Each 1 ms increase in U-RT was associated with an average increase of approximately 3% in the odds of MCI classification (95% CI 1.7%–4.6%) (Table 2).

**Table 2:** Results of logistic regression analysis.

	B	SE	Wald	df	*p*-value	Exp(B)	EXP(B) 95%CI	
							Lower	Upper
(Intercept)	− 19.15	4.53	17.85	1	< 0.001	–	–	–
U-RT	0.03	0.01	17.55	1	< 0.001	1.03	1.017	1.046

*SE* standard error, *CI* confidence interval, *U-RT* useful-field reaction time.

### Diagnostic performance of U-RT for identifying MCI using ROC curve analysis

**Fig. 4:**
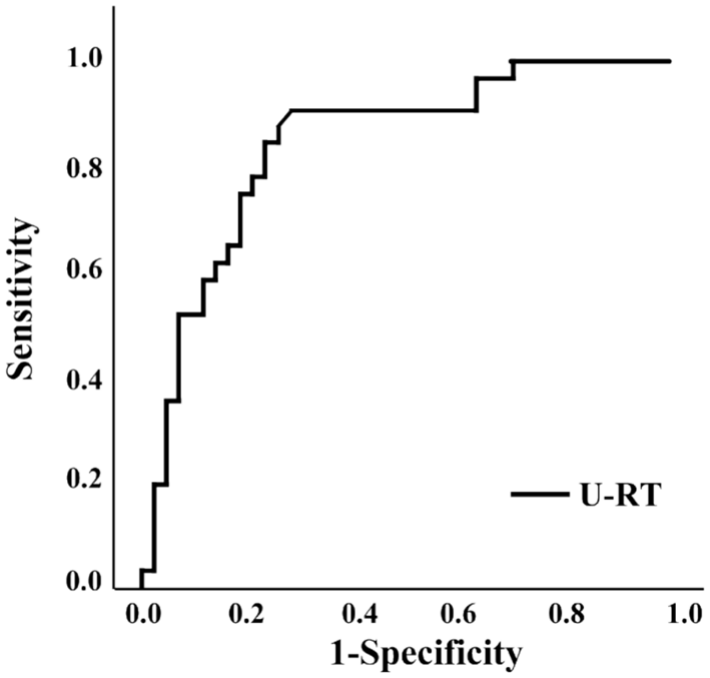
ROC curve of useful-field reaction Time (U-RT) for identifying mild cognitive impairment (MCI).

ROC curve analysis was performed to evaluate the discriminatory ability of U-RT in identifying MCI. The AUC was 0.841 (95% CI 0.741–0.916), indicating moderately accurate diagnostic performance. The optimal cutoff value for U-RT was 598.1 ms, which yielded a sensitivity of 90.3%, a specificity of 72.1%, and the maximum Youden Index of 0.624 (Fig. 4). This cutoff (598.1 ms) corresponded to a predicted probability threshold of 0.331 in the logistic regression model.

The optimal cutoff value for U-RT was 598.1 ms, with a sensitivity of 90.3%, a specificity of 72.1%, and an AUC of 0.841 (95% CI 0.741–0.916). The logistic regression model estimating the probability of MCI based on U-RT is defined as:

$${\text{logit }}\left( P \right) = - 19.15 + 0.03 \times {\mathrm{U-RT}}$$

where P represents the predicted probability of being classified as having MCI. Accordingly, the probability can be calculated as:

$$p = \frac{1}{{1 + e^{{ - \left( { - 19.15 + 0.03 \cdot {\mathrm{U-RT}}} \right)}} }}$$

This equation indicates that higher U-RT values are associated with an increased probability of MCI classification.

## Discussion

This study examined visual processing characteristics in older adults with MCI, focusing on central and peripheral (UFOV) visual tasks. We also evaluated the diagnostic utility of reaction time and gaze-related indicators using logistic regression and ROC analysis.

### Central visual processing (C-RT)

No significant differences were found in C-RT between the MCI and HC groups. This suggests that visual processing in the foveal region may remain intact in early-stage MCI. These findings align with previous research indicating that lower-level perceptual functions, such as basic visual discrimination and stimulus detection, are largely preserved during the early phases of cognitive decline^25,26^**.** Thus, central visual response latency alone may lack sensitivity for detecting early pathological changes.

### Peripheral visual attention (UFOV reaction time)

In contrast, patients with MCI exhibited significantly slower reaction times in the UFOV task, which requires broad spatial attention and rapid cognitive resource allocation. This delay likely reflects impairments in divided attention and executive function, both of which are typically affected in early MCI. The large effect size (Cohen’s d = − 1.17) underscores the robustness of this group difference. Moreover, logistic regression and ROC analysis revealed strong diagnostic utility for UFOV reaction time, with an area under the ROC curve (AUC) of 0.841 (95% CI 0.741–0.916). Using a predicted probability cut-off of 0.331, the model achieved 90.3% sensitivity and 72.1% specificity. As noted by Akobeng^27^, AUC values between 0.7 and 0.9 indicate moderately accurate diagnostic tests. These findings support UFOV reaction time as a moderately accurate and clinically meaningful behavioral marker for detecting MCI. This reinforces earlier studies on the diagnostic relevance of UFOV measures^22,28^, demonstrating that even a single behavioral metric, when properly selected, can offer substantial screening power. Neurocognitively, delayed UFOV responses may reflect early disruption in the frontoparietal attention network—particularly the dorsolateral prefrontal cortex and superior parietal lobule—regions critical for visuospatial attention and executive control. Neuroimaging studies have shown hypometabolism and functional decline in these areas in patients with MCI^29,30^, consistent with the current behavioral findings.

### Gaze-related indicators

Although gaze-related measures such as omission and commission errors, along with visual orienting frequency (VOF), did not differ significantly between groups, upward trends were observed in the MCI group. These patterns may reflect subtle impairments in inhibitory control and attentional regulation. Prior studies suggest that older adults with cognitive vulnerability exhibit greater variability in inhibitory processes and gaze regulation^31,32^. While these metrics did not reach statistical significance, they may serve as supplementary indicators if future studies incorporate more detailed eye-tracking parameters (e.g., fixation duration, saccadic latency, scan path entropy).

### Theoretical context and dual-task interference

The additional analyses reinforced that the prolonged U-RT observed in individuals with MCI cannot be attributed to general alertness or time-on-task effects. The significant Task × Group interaction showed that the group difference was especially pronounced in the UFOV condition. Moreover, even after controlling for baseline response speed using C-RT as a covariate, the group effect on U-RT remained robust. Together, these results confirm that U-RT reflects spatial-attentional demands specific to the UFOV paradigm rather than nonspecific factors such as fatigue or reduced alertness. However, it is important to note that spatial attention itself can be influenced by fluctuations in alertness. Prior work has shown that reduced alertness impairs the allocation of spatial attention^33^. Therefore, even though baseline alertness was statistically controlled in our ANCOVA, the potential modulatory effects of moment-to-moment alertness fluctuations should be considered when interpreting UFOV performance in older adults. Although the total duration differed between tasks, the additional analyses demonstrated that time-on-task or fatigue effects could not account for the selective slowing observed in the UFOV condition.

The observed correlation between C-RT and U-RT suggests that general alertness partially influences UFOV performance. However, because C-RT did not differ significantly between groups while U-RT did, alertness alone cannot explain the diagnostic value of U-RT. This indicates that UFOV RT reflects additional cognitive demands such as divided attention and executive control, which may be more sensitive to early MCI-related changes. These results are also interpretable within dual-task interference and attentional bottleneck frameworks, as originally proposed by Welford^34^ and Pashler^35^. At the same time, our findings can also be understood through the classic Posner ‘spotlight’ model of spatial attention, in which participants must disengage, move, and re-engage attention on each trial. Prior work has shown that this attentional network is disrupted in early Alzheimer’s disease^36^, supporting the interpretation that slowed UFOV RT in MCI reflects early dysfunction of the frontoparietal attention network. Considering both frameworks may provide a more comprehensive account of the attentional demands in UFOV tasks.

### Clinical implications

Given its simplicity, brevity, and scalability, the UFOV task holds promise for community-based cognitive screening. The rise of disease-modifying therapies for early Alzheimer’s disease highlights the need for early identification tools. UFOV-based assessments, especially when integrated into digital platforms, may help detect cognitive decline before functional impairments emerge. Our findings suggest that UFOV reaction time possesses sufficient sensitivity and specificity to serve as a frontline screening measure.

To further examine clinical utility, we conducted supplementary ROC analyses comparing UFOV-RT with MoCA and MMSE. UFOV-RT (AUC = 0.841) demonstrated comparable or superior discriminatory accuracy relative to MoCA (AUC = 0.812) and MMSE (AUC = 0.774) in our sample. Although UFOV assessment requires specialized equipment, these findings suggest that the additional effort may be justified by its higher sensitivity. Moreover, with ongoing advances in digital health technology, UFOV-RT tasks could be feasibly implemented in portable, tablet-based platforms for community-based screening.

Although MoCA and MMSE were used as operational criteria to classify participants in this study, direct comparisons between these tools and UFOV-RT would be problematic, as it would involve benchmarking UFOV-RT against the very measures that defined group membership. Instead, UFOV-RT provides a distinct, objective indicator of visual processing performance. Unlike conventional screening tests, which rely on subjective, paper-and-pencil responses, UFOV-RT reflects behavioral and neurocognitive mechanisms associated with the pathophysiology of MCI. Accordingly, UFOV-RT may yield unique and clinically valuable insights for the early detection of cognitive decline, beyond what is captured by traditional screening instruments.

## Limitations and future directions

First, the cross-sectional design limits causal inference and prevents evaluation of longitudinal change. Second, the modest, relatively homogeneous sample constrains generalizability. Third, MCI classification was based solely on MoCA and MMSE scores. While these are widely used screening tools, the gold standard typically involves a consensus clinical diagnosis incorporating multidisciplinary evaluation. Therefore, our classification should be considered provisional, and future studies should validate UFOV-RT against consensus diagnostic criteria. Fourth, gaze behavior metrics were limited in scope; future research should incorporate a wider range of eye-tracking variables. Fifth, the stimulus presentation software provided only the overall mean RT for the 80 UFOV trials, which precluded analysis across smaller blocks of 20 trials. Consequently, we could not directly examine time-on-task effects. Moreover, although we found a significant correlation between central RT and UFOV RT, suggesting that alertness partially influences UFOV performance, this factor did not fully account for the group differences observed. Future studies should incorporate task designs that allow more fine-grained analyses of sustained attention effects. Sixth, the visual stimulus program was designed to present peripheral targets randomly at four locations (45°, 135°, 225°, 315°). Consequently, reaction times could not be compared across individual directions, and our findings therefore reflect the overall difference between central and U-RT rather than direction-specific effects.

Lastly, tasks were administered on a 2D monitor in a controlled laboratory environment, which may reduce ecological validity. Using immersive platforms, such as virtual reality, may offer more generalizable insights into real-world attentional performance.

## Conclusion

UFOV reaction time appears to be a sensitive and valid behavioral marker for distinguishing older adults with MCI from cognitively healthy peers. Its strong diagnostic performance, confirmed by logistic regression and ROC analysis, and its classification as moderately accurate per Akobeng^27^, support its use in early detection. Longitudinal studies are warranted to assess its predictive value for dementia progression and responsiveness to intervention.

## Data Availability

The dataset generated and analyzed during the current study is available in the Mendeley Data repository at 10.17632/hnwrnk8mcw.1.
